# Residual Feed Intake in Dairy Ewes: An Evidence of Intraflock Variability

**DOI:** 10.3390/ani10091593

**Published:** 2020-09-07

**Authors:** Eliel González-García, João Paulo Dos Santos, Philippe Hassoun

**Affiliations:** 1 INRA UMR868 Systèmes d’Elevage Méditerranées et Tropicaux (SELMET), 34060 Montpellier, France; philippe.hassoun@inrae.fr; 2 Faculty of Veterinary, Universidade Federal do Pará (UFPA), Av. dos Universitários, S/n-Jaderlândia, Castanhal-PA 68746-630, Brazil; santosjp12@gmail.com

**Keywords:** feed efficiency, residual feed intake, lactating dairy ewes, intraflock variability

## Abstract

**Simple Summary:**

Few, if any, reference is available in residual feed intake in dairy sheep. In this study, carried out during more than two months with French Lacaune dairy ewes in mid-lactation, we demonstrated an intraflock variability in feed efficiency determined by, beyond litter size and daily milking frequency, evident differences between the individuals in their efficiency of using the available total mixed rations.

**Abstract:**

This study examined the intraflock variability of feed efficiency in dairy ewes, through monitoring residual feed intakes (RFI). Primiparous lactating ewes (*n* = 43; 57.7 ± 0.91 kg body weight [BW] at lambing), representative of a French Lacaune dairy flock, were allocated in an equilibrated 2 × 2 factorial design experiment, lasting for 63 days during mid-lactation and combining 2 litter sizes (singletons, SING or twins, TWIN) and 2 daily milking frequencies (once, ONE or twice, TWO). Weaning occurred, and milking started, at 35 days after lambing (DIM). Ewes were individually fed a diet based on ryegrass silage, local hay, and supplements. Individual dry matter intake (DMI) was recorded daily and further used to evaluate (and compare) differences in RFI between ewes at 42, 49, 56, 63, 70, 77, 84, 91, and 98. Average individual RFI were calculated weekly since the first week (i.e., 35–42 DIM). Total (BW) and metabolic (BW^0.75^) body weight, body condition score BCS, milk yield, and plasma non-esterified fatty acids NEFA were monitored weekly. Differences in DMI were mainly due to the lactation stage and litter size and were 11% higher in ewes with TWIN compared to SING. This was positively correlated to milk yield and consistent with differences in RFI which varied due to litter size and to the milking frequency × lactation stage interaction. Ewes that lambed SING showed higher feed efficiency (−0.08 ± 0.018 vs. 0.13 ± 0.014 kg DM/ewe/d of RFI in SING vs. TWIN, respectively), whereas there were no differences in BW or BCS. Milking frequency did not affect DMI but milk yields were higher in TWO, which was related to a higher feed efficiency in this group (0.115 ± 0.016 vs. −0.07 ± 0.016 kg DM/ewe/d of RFI in ONE vs. TWO, respectively). Average RFI was affected (*p* < 0.0001) by the ewe, thus allowing a ranking among individuals to be established. High (*n* = 22) or low (*n* = 21) feed efficiency ewes averaged −0.17 ± 0.09 or 0.18 ± 0.09 kg DM/d RFI, respectively. Estimates of RFI were not correlated to the individual milk production potential. Even if no differences in BW, BW^0.75^, or BCS were detected, high-efficiency ewes mobilized 1.5 times their body reserves (0.30 vs. 0.20 mmol NEFA/L of plasma) when compared to the low-efficiency group. The observed intraflock variability in feed efficiency of this dairy ewes’ flock was affected by litter size and milking frequency but also by evident differences between individuals’ physiologies.

## 1. Introduction

It is well known that feed accounts for most of the total farm expenses in animal production systems and that a possible solution to reduce overall feed costs and alleviate the associated negative environmental impacts is to select for feed efficiency traits. In the past, producers have primarily focused on feed conversion ratios; however, animals with similar ratios differ in feed intake and productivity. As an alternative, Koch et al. [1] proposed selecting residual feed intake (RFI), sometimes referred to as net feed intake. Considered as the deviation of actual intake from the predicted intake for a given measure of growth (ADG) and body weight, RFI can be used to compare individuals with the same or differing levels of production during the period of measurement. 

In contrast to feed conversion, selection based on RFI seems to select for lower rates of consumption and animal maintenance requirements than contemporaries to yield the same amount of product without changing adult weight or rate of gain, so theoretically, these animals should cost less to feed on a daily basis when the costs of all other maintenance factors for these animals (breeding, health, etc.) are held constant [2,3]. Heritability of RFI has been reported to be moderate, e.g., 0.06 to 0.24, depending on lactation stage [4] in dairy cattle e.g., 0.32–0.33 [5,6]. Nevertheless, five major physiological processes are likely to contribute to variations in RFI, with these processes being associated with the intake and digestion of feed, metabolism (anabolism and catabolism associated with and including variation in body composition), physical activity, and thermoregulation [7]. 

Thus, there is growing interest among producers with respect to using RFI as a tool for genetic improvement, with a greater experience in swine [8] and poultry [9], numerous research efforts have investigated the effectiveness of selecting for feed efficiency using RFI in beef cattle [10,11,12], dairy cattle [3,13,14], or sheep [15,16,17,18]. The sheep industry, however, has yet to fully investigate the potential impacts associated with selecting for RFI on carcass merit, growth traits, reproduction traits, and fleece characteristics [15]. In the particular case of the dairy sheep sector, to our knowledge, there is no available information on RFI. Beyond its economic attractiveness, evaluating factors affecting intraflock variability in feed efficiency also contributes to increasing our knowledge regarding the available spectrum of adaptive capacities that can be found at the intraflock level, when the interpretation of individual RFI is combined with other physiological processes like body reserves mobilization-accretion. 

The objective of this study was to evaluate factors affecting the intraflock variability in the feed efficiency of individually fed primiparous Lacaune dairy ewes, by focusing on the analysis of individual differences in RFI during several weeks in mid-lactation. We evaluated the hypothesis that variability in feed efficiency of individuals belonging to the same breed, cohort, productive purpose, with similar age, and reared under identical conditions, could be due to non-genetic factors but also to differences between the individuals. A second hypothesis was that mechanisms responsible for differences in RFI among individuals would probably be related to those linked to the use of body reserves. 

## 2. Materials and Methods

### 2.1. Animals, Management, Treatments, and Measurements

The experiment was carried out with a representative flock of primiparous Lacaune dairy ewes belonging to the INRAE Experimental Farm La Fage, Causse du Larzac (43°54′54.52″ N; 3°05′38.11″ E; ~800 m above sea level), Aveyron, France, following the procedures approved by the Regional Ethics Committee on Animal Experimentation, Languedoc-Roussillon (France), Agreement 752056/00.

A detailed description of the animals, management, and experimental design used for collecting the data employed in this study can be obtained from González-García et al. [19]. We used the data belonging to the primiparous (PRIM) group, considering that they were individually fed. Nevertheless, new data and information mainly related to dry matter intake (DMI) and the calculated RFI is used only for the objectives and purposes of the current work. Briefly, forty-three Lacaune dairy ewes (PRIM; two-tooth ewes) were chosen from the main flock at the end of pregnancy. Due to inconsistency in the daily individual dry matter intake measurements, five ewes were removed from the initial group (*n* = 48) participating in the experimentation reported by González-García et al. [19]. Lambing took place in January (mean lambing date was 12 January) with a mean body weight (BW) of 57.7 ± 0.91 kg. Litter size was determined about 2 months before lambing, by obtaining an ultrasound image for each ewe in the flock. The effects of two contrasting daily milking frequencies (once, ONE vs. twice, TWO) were evaluated. Approximately one half of the experimental flock was submitted to the ONE milking regime, whereas their performance was compared to the other group, which was submitted to the conventional milking frequency (TWO). 

The experimental ewes were thus distributed in homogeneous groups according to BW, body condition score (BCS), and litter size and were allocated to a 2 × 2 factorial design according to litter size (lambing singletons [SING], *n* = 16 or twins [TWIN], *n* = 27) and daily milking frequency (FREQ; ONE, *n* = 24; or TWO, *n* = 19). Thus, the design finally comprised 4 randomly assigned balanced groups for which the mean BW at lambing was as follows: (1) SING × ONE (*n* = 8; 57.2 ± 2.46 kg); (2) SING × TWO (*n* = 8; 59.7 ± 2.47 kg BW); (3) TWIN × ONE (*n* = 15; 58.5 ± 1.62 kg BW); (4) TWIN × TWO (*n* = 12; 56.1 ± 1.44 kg). 

Ewes were housed in confinement in straw-bedded pens and had access to an individual animal feeder post controlled by an electronic device that allowed each animal to get into its correct place using individual electronic identification (IDE). Each ewe was equipped with an IDE ear tag that recorded its presence at the feed bunk and allowed (or not) access to the individual feeder. When an ewe approached the feed bin, the unique passive ear transponder was identified, the barrier was unlocked, and the animal was allowed access to the feed.

Ewes were thus individually fed with a standard ad libitum lactating total mixed ration (TMR) for dairy ewes composed of a dry matter (DM) basis of 54% ryegrass silage, 6% of a second cut alfalfa-cocksfoot hay, 14% of a third harvest local hay called *Foin de Crau*, composed of a multiple mixture of grasses, legumes, and other species, 15% barley grain, 8% dehydrated alfalfa (*Luzapro*, 26.5% crude protein), and 4% commercial concentrate (*Brebitane*, 46% crude protein). The TMR was offered twice daily, one-third in the morning and two-thirds in the afternoon, at about 9 a.m. and 6 p.m., respectively. The chemical composition and nutritive value of the ingredients and TMR is presented in Table 1. The TMR distribution was adjusted to an allowance rate of 115% of the previous day’s voluntary intake. In addition, 90 g DM of *Brebitane* was offered at each milking in the milking parlor, or twice this amount in the morning for the group that was milked once a day. Ewes had free and continuous access to fresh water and salt block.

### 2.2. Determination of Residual Feed Intake (RFI) and Monitoring Related Zootechnical and Metabolic Parameters

The quantities of feed offered and refused (also analyzed for DM content) were recorded daily in order to determine the individual daily actual feed intake and thus the daily DMI per ewe. Average DMI was thus individually calculated weekly, and further used to evaluate (and compare) individual RFI per ewe. The RFI is defined as the difference between the observed or actual feed intake of an animal and its theoretical feed intake based on its requirements, as affected by its BW, production level, physiological stage, etc. Different methods for calculating RFI have been used and reported in the literature and most researchers calculate RFI by multiple regression (i.e., as proposed by Archer et al. [20]). However, we tried to calculate our RFI with this method without success. We speculate this does not fit to our objective due to the different characteristics of our experimental model (adult dairy ewes and not animal in growth). Therefore, expected feed intake was then calculated based on the equation proposed by Bocquier et al. [21], available at the *Institut National de la Recherche Agronomique* (INRA) tables recommendations for dairy sheep [22].

The equation proposed by Bocquier et al. [21] take into account the fill unit (FU) system of the French INRA system on which the ingestion capacity (IC) of a ruminant depends only on its performance. One FU corresponding to the bulk in the rumen of 1 kg of standard grass (young pasture grass with an organic matter digestibility of 77%). Indeed, supplementing basal rations with concentrated feeds will provoke reductions in the voluntary forage intake, which will be estimated by the so-called substitution rate (S). Therefore, ad libitum, the IC will be saturated by forages (F) and concentrates (C) which respectively will have their own fill values VEF (*Valeur Encombrement Fourages*) and VEC (*Valeur Encombrement Concentrés*) (VEC = S × VEF). The equation proposed for Lacaune dairy ewes by Bocquier et al. [23] considers the energy standardized milk yield (SMY) [22] and the BW of each ewe involved in the study and is as follows:

*IC* (*SFU*) = 0.024 × *BW*(*kg*) + 0.90 × *SMY*(*l/d*) (1)

where *IC* is the individual feed intake capacity (or DMI), expressed in *SFU* (fill units for sheep), and *SMY* is the energy standardized milk yield of the ewe at a given lactation stage point.

After calculating the daily average theoretical feed intake for each ewe, the RFI was then calculated as the difference between such theoretical, expected feed intake and the actual, individually measured feed intake in the experiment. Measurements of RFI were scheduled at 35, 42, 49, 56, 63, 70, 77, 84, 91, and 98 days relative to lambing (or days in lactation, DIM). For that purpose, average DMI was individually calculated weekly (i.e., once a week at each DIM) and further used to evaluate (and compare) individual RFI per ewe. That means that, every week, one average value for each of the two parameters (DMI and RFI) was calculated for each ewe based on their previously calculated daily values (e.g., for day 42, the value was the average between 35 and 42, that of 49 was the resultant for the period 42 to 49 and so on). Around each sampling date, ewes were individually weighed, and BCS measurements were performed by the same trained observer with records giving a score on a scale from 1 to 5 with 0.1 increments, adapted from the original six-point scale described by Russel et al. [24]. Blood samples were also taken from each ewe before the first meal distribution for characterizing the body reserves mobilization level of the ewe through plasma free fatty acids or non-esterified fatty acids (NEFA) profile [19].

Ewes were milked twice daily at 8 a.m. and 5 p.m. Machine milking was performed in a double-24 stall parallel milking parlor. Milk yield and milk composition (fat and protein content) were monitored and standardized milk yield (SMY) was then calculated [23].

### 2.3. Data Processing and Statistical Analyses

In the first step, the effects of major sources of variation i.e., lactation stage (35–98 DIM), litter size, milk frequency, and their first-order interactions on the main variables of interest linked to the feed efficiency of these primiparous Lacaune dairy ewes, were determined by using the PROC MIXED procedure of SAS with repeated measurements (SAS; v. 9.1.3., 2002–2003 by SAS Institute Inc., Cary, NC, USA). As the milking frequency *×* DIM interaction was not significant, it was removed from the model. The effects on RFI (kg/ewe/d) and other zootechnical parameters (i.e., DMI, RFI, and actual milk yield or SMY) were then analyzed with the following statistical model:

*Y_ijk_* = *µ* + *LitSi*_*i*_ +* Ewe*_*ij*_ + *DIM*_*k*_ + *Freq*_*l*_ + (*LitSi × DIM*)_*ik*_ + (*LitSi × Freq*)_*il*_ + *ε*_*ijk*_ (2)

where *Y_ijk_* is the response at time *k* on ewe *j* with litter size *i*, *µ* is the overall mean, *LitSi_i_* is a fixed effect of litter size *i* (*_i_* = 1–2), *Ewe_ij_* is a random effect of ewe *j* with litter size *i*, *DIM_k_* is a fixed effect of time or days relative to lambing (*DIM*; 35–98) *k*, *Freq_l_* is a fixed effect of daily milking frequency *l* (*_l_* = 1–2), (*LitSi × DIM*)_*ik*_ is a fixed interaction effect of litter size *i* with time *k*, (*LitSi × Freq*)_*il*_ is a fixed interaction effect of litter size *i* with daily milking frequency *l*, and *ε_ijk_* is random error at time *k* on ewe *j* with litter size *i*.

In a second step, and after the determination of RFI, ewes were classified into high or low feed efficiency individuals based on their distribution in this experimental population, when considering their average RFI values determined for the whole experimental period (i.e., from 35 to 98 DIM). The analysis of variance (developed in the first step), allowed the level of variation at each significant intra-factor level to be analyzed in detail with regard to the main variable of interest i.e., feed efficiency through RFI. Using the PROC RANK of SAS, the average ranking of the individual ewes for the RFI variable was established. The same procedure allowed the experimental ewes to be classified as high and low milk producers. The last allowed a relationship between the individual RFI and the SMY potential of each ewe to be established using the PROC REG of SAS. The dependency of feed efficiency from milk yield potential was thus analyzed.

In the final step, once the ewes were classified as tending to belong to the high or low feed efficiency groups, the relationships between the RFI and the average total (BW) or metabolic body weight (BW^0.75^), BCS, and plasma NEFA profile were evaluated using the PROC GLM procedure of SAS. Analyses were also carried out to contrast plasma NEFA concentration versus feed efficiency of the ewe inside each milking frequency class. The general statistical model used for this was as follows:

*Y*_*ij*_ = *µ* + *FEffic*_*i*_ + *ε*_*ij*_ (3)

where *Y_ij_* is the observation, *µ*, the population mean, *FEffic_i_,* the feed efficiency rank effect (*_i_* = 1–2; low or high), and *ε_ij_* is the residual error.

For all traits, the experimental unit was considered the ewe, as they were individually fed and included in the model as a random effect. Significance was declared at probability levels of ≤5% and comparisons between means were tested with the least squares means (LSMeans) separation procedure using the PDIFF option of SAS.

## 3. Results

The statistical significance of the lactation stage, litter size, milking frequency, and first-order interactions on DMI, milk yield, and RFI are presented in Table 2. Observed differences in DMI were mainly due to the lactation stage (DIM) and litter size effects, but not because of changes in milking frequency per se. The effects of milking frequency on DMI depended on its interaction with litter size. Similar to milk yields, RFI was strongly affected (*p* < 0.0001) by the three major sources of variation evaluated here (i.e., DIM, litter size and milking frequency), and by the interaction milking frequency × lactation stage. A similar tendency to that found for milk yield was observed for the energy-corrected milk (SMY; Table 2).

Average DMI during the evaluated mid-lactation period was 11% higher in ewes that lambed TWIN when compared to those lambing SING, and was positively correlated (data not shown) to the total or SMY milk yields (Table 3). Differences (*p* < 0.0001) in RFI were also found between ewes that lambed SING and TWIN (−0.08 ± 0.018 vs. 0.13 ± 0.014, respectively; Table 3).

Milking frequency did not affect DMI as an isolated factor, but rather, there was a significant interaction with litter size (Table 2). As expected, the actual or SMY milk yields were higher in ewes being milked twice (Table 3), which was related to a higher overall feed efficiency in this group for the whole experimental period, as interpreted by differences in RFI (0.11 ± 0.016 vs. −0.07 ± 0.016 in ewes milked once vs. twice a day, respectively; Table 3).

**Figure 1:**
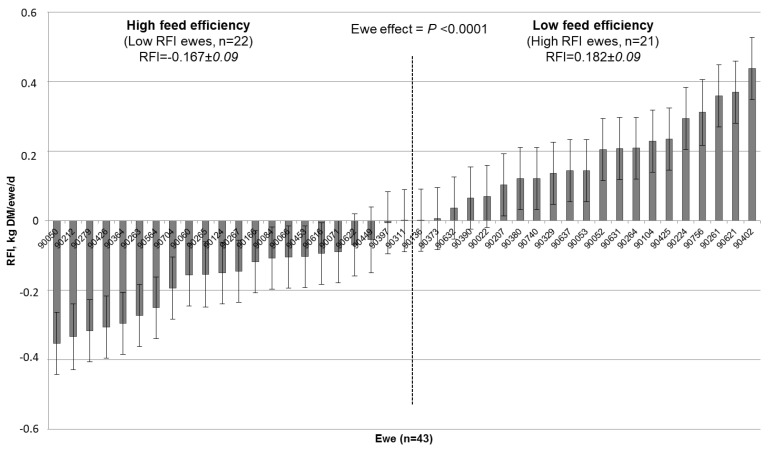
Ranking of primiparous Lacaune dairy ewes (*n* = 43) in function of their average residual feed intake (RFI, kg DM/ewe/d) during mid-lactation (42–98 days in milk). Overall RFI during the whole period was 0.004 ± 0.090 kg DM/ewe/d.

The average RFI for the whole experimental period was significantly (*p* < 0.0001) affected by the individual ewe. As a consequence, ewes were ranked as having a tendency of high or low feed efficiency according to their average RFI (Figure 1). Ewes classified as highly efficient (*n* = 22) averaged −0.17 ± 0.09 kg DM/d of RFI; whereas, on the other hand, ewes classified as low feed efficient (*n* = 21) showed an average RFI value of 0.18 ± 0.09 kg DM/d.

**Figure 2:**
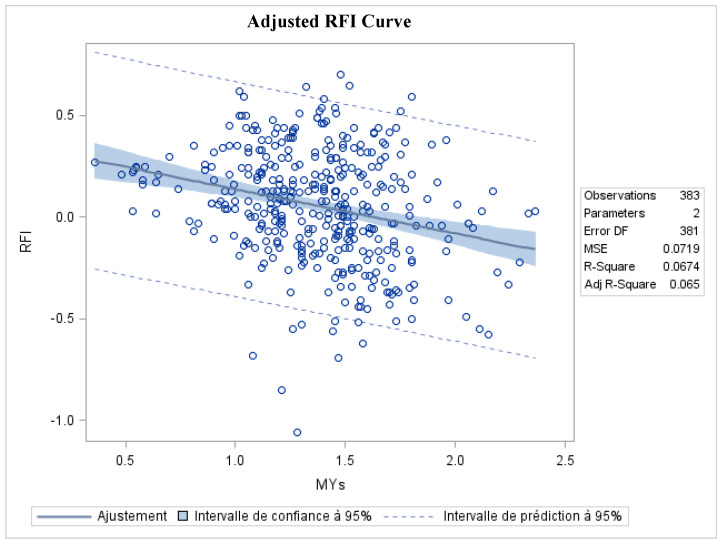
Adjusted curve for average individual residual feed intake (RFI) and fat-corrected milk yield of primiparous Lacaune dairy ewes (*n* = 43) during mid-lactation (42–98 days in milk). MYs = standardized milk yield (SMY).

The expected RFI was independent of the individual milk production potential (Figure 2). In more than half of the cases, ewes classified as tending to be high feed efficiency ewes (left side panel of Figure 1) corresponded to ewes submitted to two milking per day (13 ewes in TWO and 9 in ONE vs. 6 ewes in TWO and 15 in ONE in high vs. low feed efficiency groups, respectively; Table 4). 

**Figure 3:**
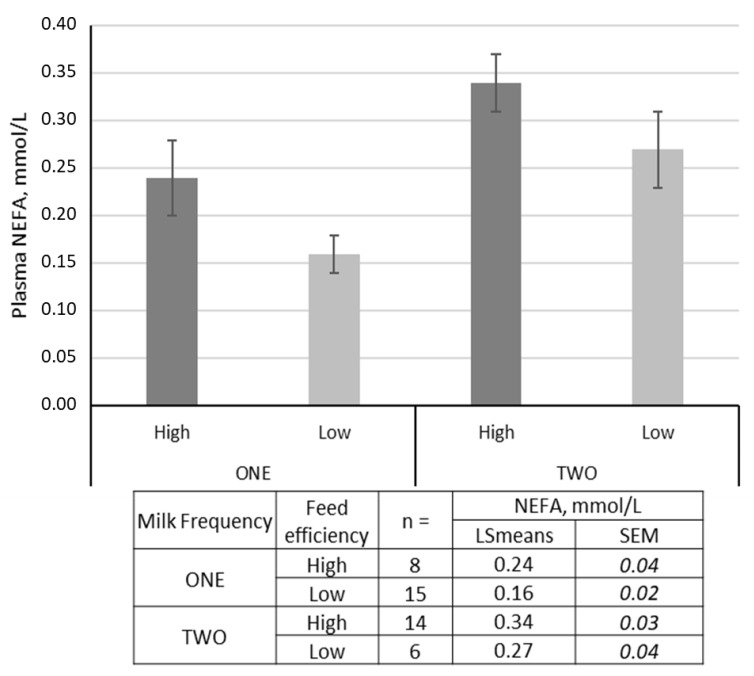
Relationships between the daily milking frequency to which the ewes were subjected (ONE vs. TWO), the ewes’ feed efficiency classification (High vs. Low) and the body reserves mobilization illustrated by the plasma non-esterified fatty acids (NEFA) concentration.

Interestingly, and even if no differences in BW, BW^0.75^, or BCS were detected, high-efficiency ewes mobilized almost two-fold their body reserves when compared to the low-efficiency group (see and compare plasma NEFA values in Table 3). As expected, ewes milked once a day presented lower plasma NEFA than ewes milked twice (0.2 ± 0.03 vs. 0.3 ± 0.04 mmol/L). Either in ONE or TWO ewes, high-efficient ewes mobilized more BR compared to low-efficient ewes (Figure 3).

## 4. Discussion

Evaluating factors affecting intraflock variability of feed efficiency, through RFI, increases our knowledge regarding the available spectrum of adaptive capacities which can be found at the intraflock level. This becomes more interesting when the interpretation of RFI is combined with other physiological processes such as body reserves mobilization-accretion processes. However, the exercise is also interesting from an economic point of view for the industry in question since the identification of animals that require less feed for equivalent production would clearly increase overall farm productivity, thus leading to the argument that feed conversion efficiency of farm animals could be considered an important component of the profitability of farming systems [14,15,17,25].

The RFI quantifies efficiency within a production level and allows for the identification of animals that convert gross energy into net energy more efficiently by reducing energetic losses in feces, urine, gas, and non-maintenance heat; thus, this is independent of the dilution of maintenance when multiple of maintenance is calculated based on requirements for observed production [3].

Currently, there are several reports arguing the interest, pertinence, and possibilities of using RFI as a selection characteristic to increase feed efficiency and farm profitability in non-ruminants [8], but also in ruminants (beef: [10,11]; dairy: [3,13,14]). There is a lack of information, however, in small ruminants, although some works have been developed mainly during the growth phase in sheep [14,15,16,18] and the sheep industry has yet to fully investigate the potential impacts associated with selecting for RFI on carcass merit, growth traits, reproduction traits, and fleece characteristics [15].

In the dairy sheep industry, to our knowledge, there is no available information on RFI studies. Our work and results could thus be considered original in that sense. The question of using RFI as a tool for increasing feed efficiency in the future dairy ewe’ industry remains.

Here, we evaluated different factors with the potential for affecting feed intake i.e., litter size at lambing and during the suckling period, milking frequency, and lactation stage. However, we were also able to confirm evidence for individually intrinsic factors leading to differences in feed efficiencies at the intraflock level in ewes belonging to the same dairy breed, cohort, with the same age and reared under identical conditions, using the same day-to-day management and feeding.

Differences between the energy requirements of ONE and TWO milking frequencies and ewes lambing SING or TWIN were great enough to cause significant differences in energy partitioning, which were translated into differences in milk yield and feed intake. Thus, our findings provide support for the use of RFI as a tool to identify animals that eat less than others within a production level, independent of the energy balance or the related management practice.

Although some re-ranking occurred throughout the experimental period, this was minor. This allows to conclude that within a level of production, ewes that eat less when receiving a particular management (e.g., milked once daily) should also consume less than their contemporaries when returning to the average management of the flock (e.g., being milked twice a day).

We also verified that RFI was independent of the individual milk production potential (Figure 2). Thus, we could assume that ewes with low RFI required less feed to produce the same amount of milk as their contemporaries, independently of their milk production potential. Consistent with these results, the most feed-efficient ewes (*n* = 22) ate ~350 g of DM/d less than the least efficient ewes in our study (i.e., −0.17 ± 0.09 vs. 0.18 ± 0.09 kg DM/d of RFI in high and low feed efficiency ewes, respectively).

Potts et al. [3] reported that RFI was moderately repeatable across 2 consecutive feeding periods for replacement of beef heifers classified as high (>0.5 SD), medium (±0.5 SD), and low (<−0.5 SD) RFI. These authors argued that the estimation of RFI across different periods may be more repeatable if measurements are obtained from periods when animals were in similar physiological stages. There is little to no information available on those effects in cattle or sheep, but species may differ in their response to RFI selection [15].

Here, we focused on the mid-lactation of this Lacaune dairy flock, an important period on which a relative stabilization is achieved; thus, comparison among individuals becomes feasible. The weekly estimates of RFI were also repeatable within and across the group of ewes in the design, which suggests that we were able to account for many of the production and BW changes that occurred from one week to the next, affecting the calculation of RFI. However, genotype × environment interactions may be an important factor to take into account.

While upwards of 60 days of feed intake measurements are needed to accurately estimate RFI in beef cattle [26], the necessary duration in sheep is unknown [15]. In our study, we chose a period (63 days) similar to that recommended by Sainz and Paulino [26] which also fits well in the range indicated by Cockrum et al. [15] (42–63d) for determining RFI in sheep. The last authors confirmed that both the variance and the R^2^ of their results in rams provide support that a period of 40–63 days is needed to accurately determine individual RFI values in sheep.

Furthermore, the current study contributes to the literature on the relationship between RFI and productive performance in dairy ewes offered a forage diet. To date, the majority of studies examining this area in sheep have focused on growing or finishing animals offered energy-dense diets.

Similar to reports in beef cattle, our RFI results varied widely in dairy ewes (see standard errors in Figure 1). This variation may be attributed to individual differences in heat production from metabolic processes, body composition, and physical activity; these factors account for around 70% of the variation in RFI [7], but were not measured in this study.

Results indicated that BW, BCS, and milk production potential are phenotypically independent of RFI estimates. Further research is necessary however to determine the relative weighting value for RFI in successive physiological stages before it can successfully be considered a potential selection tool.

Based on their results, Cockrum et al. [15] recommended that, ideally, selection decisions for RFI in sheep should take place at weaning, and feed efficiency status should be applicable over an animal’s lifetime.

Fitzsimons et al. [10] found changes in backfat thickness, which were negative for low-RFI cows and positive for high-RFI cows. The reduction in backfat thickness in low-RFI (efficient) cows suggested that these cows were mobilizing more body fat to meet their nutritional requirements during pregnancy than high-RFI cows. These authors also suggested that the calculation of RFI in beef cows should include body composition traits such as subcutaneous body fat and BCS.

Potts et al. [3] also argued that because body energy changes are accounted for in the prediction of RFI, it is expected that cows with low RFI will not be any more likely to mobilize body tissue to support production than cows with high RFI. The independence of RFI from BW loss is important because excessive tissue mobilization can lead to negative energy balance, which is related to metabolic diseases and poor fertility.

Our results are in agreement with statements relating feed efficiency with body reserves utilization. We found a negative correlation between RFI and the profile of body reserves mobilization, analyzed through regular monitoring of plasma NEFA, with the low-RFI (high-efficiency) ewes consistently showing higher plasma NEFA. Even if no differences in BW, BW^0.75^, or BCS were detected, high-efficiency ewes mobilized almost two-fold their body reserves when compared to the low-efficiency group. Irrespective of the milking frequency, ewes qualified as high feed efficient mobilized more body reserves than those qualified as inefficient. The latter probably supported a higher energy requirement for milk production, considering the larger proportion of ewes being milked twice (TWO) in the high-efficiency group (Figure 3). However, three of the four most efficient ewes lambed singletons and were milked once a day. In the available literature, we did not find any previous report concerning the direct relationships between efficiency in body reserves administration and RFI in small ruminants and particularly in dairy sheep, as evidenced in the current study.

## 5. Conclusions

Under the conditions of this experiment, low-RFI lactating dairy ewes ate less and produced milk at similar levels when compared to high-RFI cohorts, without changing body weight or BCS; thus, they could be said to use their feed more efficiently. The observed intraflock variability in feed efficiency is probably the consequence of indirect factors affecting metabolism and energy balance of ewes, like litter size and milking frequency, but it is also affected by differences among the implicated individuals’ mechanisms non-elucidated here. However, entry into different physiological stages may present some challenges and more research will be needed to investigate the long-term applicability of the RFI estimates found here. Finally, this is probably the first report demonstrating a close relationship between RFI and body reserves mobilization in small ruminants, and particularly in dairy ewes.

## Figures and Tables

**Table 1:** Nutritive value of ingredients and total mixed ration (TMR).

Diet Ingredients and TMR	% In TMR	Organic Constituents, g/kg DM ^1^						Digestibility, %		Net Energy, /kg DM	Protein Value, g/kg DM		Fill Value
		OM ^2^	CP ^3^	CF ^4^	NDF ^5^	ADF ^6^	ADL ^7^	DMD ^8^	OMD ^9^	UFL ^10^	PDIN ^11^	PDIE ^12^	SFU ^13^
Ryegrass silage	54	907	144	486	317	282	36	64.4	60.8	0.81	87	74	1.41
Alfalfa-cocksfoot hay	5.5	934	169	492	315	296	68	62.9	59.8	0.71	108	94	1.15
Mixed hay	13.5	902	117	526	318	282	54	60.3	57.1	0.72	73	79	1.33
Barley grain	8	927	264	365	176	147	66	75	71.2	0.99	168	139	-
Dehydrated alfalfa	15	877	86	128	31	26	7	92.3	90.8	1.12	80	101	-
Concentrate	4	921	460	264	140	122	43	86.3	83.7	1.15	391	368	-
TMR	100	94	157	419	255	227	39	70	67	0.87	104	98	1.01

^1^ DM = Dry matter content; ^2^ OM = Organic matter content; ^3^ CP = Crude protein content; ^4^ CF = Crude fiber content; ^5^ NDF = Neutral detergent fiber content; ^6^ ADF = Acid detergent fiber content; ^7^ ADL = Acid detergent lignin content; ^8^ DMD = Dry matter digestibility; ^9^ OMD = Organic matter digestibility; ^10^ UFL = net energy for milk production, 1 UFL = 1700 kcal; ^11^ PDIN = PDIA (dietary protein undegraded in the rumen which is digestible in the small intestine) + PDIMN (microbial protein that could be synthesized from the rumen degraded dietary N when energy is not limiting); ^12^ PDIE = PDIA + PDIME (microbial protein that could be synthesized from the energy available in the rumen when degraded N is not limiting); ^13^ SFU = Fill unit for sheep.

**Table 2:** Significance (*p*-value) of the fixed effects lactation stage (days in milk, DIM), litter size (LS), milking frequency (MF), and their interactions on residual feed intake (RFI, kg/ewe/d) and other zootechnical parameters linked to feed efficiency of primiparous Lacaune dairy ewes during lactation (42–98 DIM).

Items	DIM	Litter Size	Milking Frequency	First-Order Interactions across Major Fixed Effects			
				LS × MF	LS × DIM	MF × DIM	LS × MF × DIM
DMI	0.0121	<0.0001	0.1604	0.0142	0.9841	0.9937	0.9184
MY	<0.0001	<0.0001	<0.0001	0.0224	0.9970	0.9740	0.9843
SMY	<0.0001	0.0168	<0.0001	0.0015	0.9692	0.9322	0.9751
RFI	<0.0001	<0.0001	<0.0001	0.9837	0.4963	<0.4177	0.6669

**Table 3:** Effects of litter size and milking frequency on dry matter intake (DMI, kg/ewe/d), actual or standardized (SMY) milk yields (L/ewe/d), and residual feed intake (RFI, kg/ewe/d) of individually fed primiparous Lacaune dairy ewes at mid-lactation (42–98 DIM).

Items	Litter Size		Milking Frequency	
	SING	TWIN	ONE	TWO
Dry matter intake (DMI, kg/ewe/d)	2.15 ± 0.029	2.39 ± 0.023	2.29 ± 0.026	2.24 ± 0.027
Milk yield (kg/ewe/d)	1.55 ± 0.032	1.71 ± 0.025	1.50 ± 0.028	1.76 ± 0.030
Standardized milk (SMY, L/ewe/d)	1.33 ± 0.024	1.41 ± 0.019	1.26 ± 0.021	1.48 ± 0.022
RFI (kg DM/ewe/d)	−0.08 ± 0.018	0.13 ± 0.014	0.11 ± 0.017	−0.07 ± 0.016

**Table 4:** Relationships between residual feed intake (RFI) and average total (BW) or metabolic body weight (BW^0.75^), body condition score (BCS), and plasma non-esterified fatty acids (NEFA) profile in individually fed primiparous Lacaune dairy ewes at mid-lactation (42–98 DIM). Ewes were classified as presenting high or low feed efficiency in accordance to their average RFI during the 8-week period.

Items	Feed Efficiency of Ewes, According to RFI Ranking				Effect, *p*-Value
	High ^a^		Low ^b^		
	LSmean	S.E.M. (±)	LSmean	S.E.M.	
BW, kg	59.0	1.46	56.3	2.81	NS
BW^0.75^, kg	21.3	0.39	20.5	0.99	NS
BCS, 1–5	2.88	0.035	2.77	0.132	NS
NEFA, µmol/L	0.39	0.017	0.22	0.016	<0.0001

^a^ High-efficiency ewes (*n* = 22) were composed by 13 and 9 ewes lambing singletons and twins, respectively. They were milked once (*n* = 9) or twice (*n* = 13) daily. ^b^ Low-efficiency ewes (*n* = 21) were composed by 3 and 18 ewes lambing singletons and twins, respectively. They were milked once (*n* = 15) or twice (*n* = 6) daily. NS = non-significant; LS = litter size; MF = milking frequency.
